# Studies on Apigenin and Its Biological and Pharmacological Activity in Brain Disorders

**DOI:** 10.34172/apb.2022.068

**Published:** 2022-01-03

**Authors:** Vishal Kumar, Satyabrata Kundu

**Affiliations:** Department of Pharmacology, ISF College of Pharmacy Moga, Punjab 142001 India.

## Dear Editor,

Apigenin (4′,5,7-trihydroxyflavone) is a dietary flavonoid that is abundantly present in many fruits, medicinal herbs, vegetables and formally belongs to the flavone sub-class.^1^ The best sources of apigenin are parsley, chamomile, celery, artichokes, and oregano.^2^ Apigenin is a compound with low toxicity and multiple beneficial bioactivities. Apigenin has several beneficial effects as an antioxidant, anti-inflammatory, blood pressure reduction, and chemo-preventive.^3^ Apigenin has an effect in the downregulation of IL-1β and TNF-α; also, it shows anti-inflammatory properties by attenuating the expression of COX-2 and iNOS.^1^ At the cellular level, apigenin acts as an inhibitor of several protein-tyrosine and serine-kinases.^3^ Apigenin has many pharmacological roles as antiphlogistic, antispasmodic, and antibacterial agent, anti-asthmatic, anti-parkinsonism agent (Table 1).^4^

**Table 1:** Recent studies on apigenin and its biological and pharmacological activities

**S. No.**	**Key outcomes**	**References**
1	Apigenin shows a protective effect in the preclinical model of down syndrome, via reduction of oxidative stress and activation of proliferative and pro-neurogenic genes (KI7, Nestin, Sox2, and PAX6). Apigenin may be a potential therapeutic candidate for the management of down syndrome.	^5^
2	Apigenin reversed decreased cell viability, the activity of sodium pump, increased LDH release, and apoptotic rate in OGD/R injury in rat hippocampal neurons. The finding suggested that it can be a novel therapeutic candidate to improve sodium pump activity.	^6^
3	In the combination of apigenin and trolox, apigenin shows a strong inhibitory effect in H_2_O_2_ induced ROS production in RAW264.7 cells as well as free radical-induced oxidative damage in erythrocytes than trolox. However, apigenin also exerts a strong inhibitory effect on LPS induced NF-kB/NLRP3/caspase-1 signaling in RAW246.7 cells than trolox. Results suggested that apigenin might be a potent drug candidate for the management of oxidative stress and inflammatory diseases.	^7^
4	AP-SD prospers the nuclear translocation of Nrf2 and increases the expression of Nrf2 as well as target genes HO-1 and NQO-1. It also enhanced the activity of SOD and GSH-Px, and decreased the level of ROS and MDA in a mouse model of AMD. The finding suggested that AP-SD could be an effective compound for the treatment of AMD.	^8^
5	Apigenin and luteolin resist the activation of astrocytes and inhibit the protein and mRNA expression in LPS-induced astrocyte cultured neurons. Apigenin also inhibits the IL-31 and IL-33 via suppression of ERK, NF-kB, and STATE. Similarly, luteolin inhibits IL-31 via suppression of JNK, p38, ERK, NF-kB, and STAT3 in astrocytes. The finding suggested that both apigenin and luteolin have the potential to treat diseases involving astrocyte activation.	^9^
6	Apigenin increased Nrf2 nuclear translocation, GSK-3β phosphorylation, and reduced apoptosis, decrease LDH release, and promote cell viability in both OGD/R cell cultures and a rat model of ischemic-reperfusion. The study suggested that apigenin could be a strong neuroprotective drug candidate.	^10^
7	Apigenin significantly delayed peripheral nerve degeneration via inhibiting of degradation of myelin and peripheral axons, and also inhibits the proliferation of Schwann cells. Thus, apigenin can be a novel therapeutic choice for treating peripheral neurodegenerative diseases.	^11^
8	Apigenin rescues memory deficits and decreases cell viability in hilus, however, it also decreases the release of cytochrome c in the kainic acid-induced rat model of temporal lobe epilepsy. The study suggested that clinically apigenin could reverse memory impairment via anticonvulsant and neuroprotective activity.	^12^
9	Apigenin significantly fettles spatial working memory and decreased degenerative neurons in hilus via complete blockade of caspase 9 and cytochrome c release in Aβ 25-35 induced rat model of Alzheimer disease. The finding suggested that apigenin can rescue the spatial working memory and neuronal degeneration via reversal of mitochondrial dysfunction.	^13^
10	Apigenin decreases oxidative stress, levels of IL-6, TNF-α, mitochondrial-mediated neuron apoptosis and also downregulates TLR4/NF-kB signaling pathways in the acrylonitrile rat model of neuroinflammation. Results suggested that it could be a potent neuroprotective agent.	^14^
11	Apigenin rescues the behavioral impairments, cognitive deficits and increases the level of BDNF, cAMP, and CREB without altering seizure severity in pentylenetetrazole kindling associated behavioral and cognitive impairments in the mouse model. However, it also increases the serotonin level in the brain. The finding suggested that apigenin may be a potent therapeutic candidate to treat memory impairment and related diseases.	^15^
12	Apigenin shows a protective effect via detracting autophagy and apoptosis in the brain against ischemia/reperfusion injury. In-vivo results show apigenin significantly decreases neurobehavioral score and increases cell proliferation by the up-regulation of VEGFR22/CD3 and affecting caveolin-1, VEGF, eNOS expression in brain tissue of MCAO/R rats.	^16^
13	Apigenin promotes the upregulation of NF-kB gene expression and inhibits the release of pro-inflammatory cytokines IL-l, TNF-α, and also prevents the reduction of BDNF and GDNF levels in rotenone-induced rat model of PD. Thus, the results suggested that apigenin could serve as an effective agent for the management of PD.	^17^
14	Apigenin rescues the antioxidant machinery via the reduction of ROS levels, prevention of activation of stress kinase (IKKβ), JNK, and activation of NF-kB in high fat-high fructose diet induce rat model of hippocampal derangements. Results suggested that it has better antioxidant potential.	^18^
15	Apigenin recovers cognitive function via restoration of histone acetylation, BDNF signaling, and suppression of pro-inflammatory cytokines and NF-kB signaling pathway in an aged rat model of isoflurane-induced cognitive dysfunction. Thus, the study suggested that apigenin can be a potential drug candidate for the treatment of post-operational cognitive dysfunction.	^19^
16	Apigenin improves cognitive impairments in the rat model of post-stroke cognitive deficits, through decreased HDAC content, up-regulation H3 and H4 acetylation in the hippocampus and increased the level of BDNF in dose-dependent manner.	^20^
17	Apigenin exhibits protective effect via reduction of MPO, ROS, MDA, and increases the level of oxidized glutathione (GSSG), GSH, hydrogen peroxide, and SOD in a rat model of SAH. Thus, the study suggested that apigenin may be considered as a potential drug for the management of SAH.	^21^
18	Apigenin exerts a neuroprotective effect against cerebral ischemic/reperfusion injury by promoting cell viability, cell proliferation and by reducing apoptotic cell death. The study revealed the neuroprotective effect of apigenin which is possibly induced by the STATE3 phosphorylation-mediated Mn-SOD up-regulation.	^22^
19	Apigenin significantly increased retention of immune cells in the periphery and decreased expression of α4 integrin and CLEC12A on splenic dendritic cells in the autoimmune encephalomyelitis mice model. The study suggested that it could be a better treatment for the management of multiple sclerosis as compared to available treatment.	^23^
20	Balez et al revealed that apigenin rescues the neurons from neuroinflammation, neuronal excitability, and apoptosis in the human induced pluripotent stem cell model of AD via the down-regulation of cytokines and nitric oxide release. These findings highlighted that apigenin could be a better therapeutic strategy for the management of AD.	^24^
21	Apigenin mitigates neuroinflammation and astrocytes integrity by the downregulation of IL-6, IL-1β expression, and upregulation of IL-10 in LPS induce *in vitro* model of neuroinflammation associated with Alzheimer disease. Thus, the finding suggested that apigenin could be a potential agent for the treatment of neurodegenerative diseases.	^25^
22	Apigenin shows a protective effect by the up-regulation of PPARγ expression, oxidative stress, inhibition of microglia, and NLRP3 activation, which subsequently down-regulate the production of IL-1β and IL-18 in a chronic unpredictable mild stress rat model of depression. Results suggested that it may be beneficial for the management of depression.	^26^
23	Apigenin reverses the decreased level of Bcl-2 and Bid, loss of mitochondrial transmembrane potential, increase levels of Bax and p53, the release of Cytochrome c, activation of caspases (3, 8, and 9), and cleavage of PARP-1 in proteasome inhibitor induce neuronal apoptosis in both PC12 and SH-SY5Y cell lines.	^27^
24	Apigenin significantly decreases early brain injury such as BBB disruption, brain edema, and cell apoptosis via the repression of NF-kB, TLR4, and their pro-inflammatory cytokines in the cortex in a rat model of subarachnoid hemorrhage. The finding suggested that apigenin could be protective against early brain injury.	^28^
25	Apigenin elevates body weight, improves cognitive dysfunction, reduces blood glucose, MDA content, and increases SOD level in the cerebral cortex and hippocampus in the streptozotocin-induced rat model of diabetes-associated cognitive dysfunction. Finding suggested that apigenin could be an effective therapeutic agent for diabetes-associated cognitive decline in rats via the suppression of apoptotic, nitric oxide, and oxidative stress pathways.	^28^
26	Apigenin rescues from the OGD/R induced neuron injury by the suppression of cell apoptosis, lactate dehydrogenase, and intracellular ROS level in PC12 cells. These results suggested that apigenin could be a therapeutic candidate against neuronal death.	^29^
27	Apigenin and luteolin both in combination rescue the dopaminergic neurons through reducing microglial activation, neuroinflammation, oxidative stress as well as enhancement of BDNF in MPTP induced mice model of PD. The study suggested that both the molecules could be potential therapeutics in PD.	^30^
28	Apigenin restores AD associated learning and memory impairment via the suppression of the amyloidogenic process, alleviating the Aβ burden, restoring ERK/CREB/BDNF pathway, and through prevention of oxidative stress in APP/PS1 double transgenic mouse model of AD. The finding suggested that apigenin could be an alternative agent for the prevention of AD-associated symptoms.	^31^
29	Apigenin exerts neuroprotective effect via maintaining redox balance by increasing cellular superoxide dismutase, glutathione level, and reduced ROS generation; obstructing p38 MAPK, MAPKAP kinase-2, heat shock protein 27 and c-jun N-terminal signaling pathways and reduced neuronal apoptosis in a copper-mediated β-amyloid neurotoxicity cell model of AD. The finding suggested that it could be a potential therapeutic for AD.	^32^
30	Apigenin exhibits a protective effect via the up-regulation of SOD, GSH-Px activity, decrease serum level of IL-1β and TNF-α, and shows antioxidative, anti-inflammatory, and anti-apoptotic properties in modified weight-drop method induced rat model of spinal cord injury.	^33^
31	Apigenin elicits neuroprotective effect through suppression of excitotoxicity in dose-dependent manner, ROS generation and reduction of GSH in hippocampal neurons in kainic acid induce an *in vitro* and *in vivo* model of excitotoxicity.	^34^
32	Apigenin shows a neuro-protective effect in the *Drosophila* model of PD through increasing GSH, dopamine content, life span and reducing the level of GST activity, MAO, lipid peroxidation, and caspase 3/9 in a dose-dependent manner. The study highlighted the neuroprotective potential of apigenin in PD.	^35^

Abbreviations:OGD/R, oxygen and glucose deprivation/reperfusion; ROS, reactive oxygen species; LPS, lipopolysaccharide; NF-κB, Nuclear factor kappa B; AP-SD, Solid dispersion of apigenin; SOD, superoxide dismutase; GSH, glutathione; MDA, malondialdehyde; AMD, age-related molecular degeneration; TNF, tumor necrosis factor; IL-6, interleukin-6; eNOS, endothelial nitric oxide synthase; MCAO/R, middle cerebral artery occlusion/reperfusion; IKKβ, Inhibitor of nuclear factor kappa-B kinase subunit beta; MPO, myeloperoxidase; SAH, subarachnoid hemorrhage; PD, Parkinson’s disease; AD, Alzheimer disease; GST, glutathione-S-transferase; MAO, monoamine oxidase.

## Acknowledgments

 The authors express their gratitude to Chairman, Mr. Praveen Garg and Director, Dr. G. D. Gupta, ISF College of Pharmacy Moga, Punjab, India for their excellent vision and support.

## Ethical Issues

 Not applicable.

## Conflict of Interest

 The authors declare that there is no conflict of interest.
